# Analysis on factors behind sentinel lymph node metastasis in breast cancer by color ultrasonography, molybdenum target, and pathological detection

**DOI:** 10.1186/s12957-022-02531-3

**Published:** 2022-03-08

**Authors:** Aibibai Yiming, Muhetaer Wubulikasimu, Nuermaimaiti Yusuying

**Affiliations:** Department of General Surgery, The Second People’s Hospital of Kashgar, Room 3 Building 3, No. 1 Sagelamu Road, Kashgar, Xinjiang, 844000 China

**Keywords:** Breast cancer, Sentinel lymph node, Molecular pathology, Factors

## Abstract

**Background:**

This study aimed to identify the factors underlying the metastasis of breast cancer and sentinel lymph nodes and to screen and analyze the risk factors of sentinel lymph node metastasis to provide a reference and basis for clinical work.

**Methods:**

A total of 99 patients with breast cancer were enrolled in this study. These patients received treatment in our hospital between May 2017 and May 2020. The general information, characteristics of the color Doppler echocardiography, molybdenum, conventional pathology, and molecular pathology of the patients were collected. Factors influencing sentinel lymph node metastasis in breast cancer patients were retrospectively analyzed.

**Results:**

In this study, age, tumor diameter, BI-RADS category, pathology type, expression profiles of CK5/6, EGFR, and CK19, and TP53 and BRAC1/2 mutations were independent risk factors for sentinel lymph node metastasis in breast cancer (*P* < 0.05). The number and locations of tumors, quadrant of tumors, regularity of tumor margins, presence of blood flow signals, presence of posterior echo attenuation, presence of calcification, histological grade, molecular typing, and mutations of BRAF, ATM, and PALB2 were irrelevant factors (*P* > 0.05).

**Conclusions:**

In conclusion, age, tumor diameter, BI-RADS category, invasive type, expression of CK5/6, EGFR, and CK19, and mutations in TP53 and BRAC1/2 were positively correlated with sentinel lymph node metastasis. These independent risk factors should be given more attention in clinical studies to strengthen the management and control of sentinel lymph node metastasis in high-risk breast cancer and support early chemotherapy or targeted therapy.

## Background

Breast cancer is a malignant tumor that commonly occurs in the lactiferous ducts and mammary gland epithelium. Currently, it has the highest incidence rate of lung cancer worldwide [1]. The sentinel lymph node is considered as the first met lymph node in the metastasis process of tumors from tumor-draining areas, and it builds the initial barrier for tumor metastasis. Sentinel lymph node biopsy is widely used for breast cancer operations [2], which can reduce the incidence of complications such as upper extremity lymphedema, limb numbness, and limited shoulder motion caused by axillary lymph node dissection. Thus, it gradually replaces total axillary lymph node dissection as the “gold standard” for axillary management in patients with early breast cancer [3, 4]. Metastasis of sentinel lymph nodes in breast cancer is directly related to the clinical stage. It is of great significance to detect it in time to improve the clinical cure rate and prognosis of breast cancer patients [5]. Although a correct axillary demarcation may preserve the axillary lymph nodes as much as possible in breast surgery, patients with higher BMI and increased axillary disease burden and axillary radiation may have higher risks of developing arm lymphedema [6]. Prolongation of survival in patients with breast cancer due to early diagnosis and modern methods of treatment has turned the attention to risk factors underlying the metastasis of sentinel lymph nodes, which is the most important morbidity secondary to the treatment of the disease [6–8]. Currently, there are many clinical models for the prediction of sentinel lymph node metastasis, among which color Doppler ultrasound, molybdenum target, and pathological biopsy are the main options. However, because of the different prediction abilities of cancer centers around the world, it is necessary to construct a predictive system that is suitable for primary hospitals in China [9, 10]. By collecting general information, the characteristics of the color Doppler echocardiography, molybdenum, pathological, and molecular pathological data from 99 breast cancer patients, we analyzed the risk factors affecting sentinel lymph node metastasis in a bid to filter out the risk factors, providing a reference and basis for clinical studies.

## Database and method

### General data

A total of 99 patients with breast cancer were enrolled in this study. These patients received treatment in our hospital between May 2017 and May 2020. They were divided into two groups according to whether the sentinel lymph nodes in frozen pathology were metastatic during radical mastectomy or subtotal mastectomy, with 49 or 50 patients in each group. The general information of the two groups is presented in Table 1.

**Table 1:** Univariate logistic regression analysis of general data of the two groups of patients

General information	Classification	Lymph node metastasis group (*n* = 49)	Non-lymph node metastasis group (*n* = 50)	*Z/χ*^*2*^	*OR*	*P* value
Age		62 (47–78)	53 (45–72)	3.74	1.24 (1.07~1.41)	0.04
Tumor diameter (cm)		8 (4–8)	4 (2–8)	5.77	1.45 (1.26~1.63)	0.02
Tumor number (%)	Single	40 (81.63%)	43 (86%)	0.13	0.93 (0.41~1.30)	0.94
	Multiple	9 (18.37%)	7 (14%)			
Tumor location (%)	Left	28 (57.14%)	26 (52%)	0.24	0.98 (0.93~1.07)	0.77
	Right	21 (42.86%)	24 (48%)			
Tumor quadrant (%)	Outside and up	26 (55.06%)	25 (50%)	0.29	0.91 (0.52~1.24)	0.77
	Outside and down	7 (14.29%)	8 (16%)			
	Inside and up	13 (26.53%)	15 (30%)			
	Inside and down	2 (4.08%)	1 (2%)			
	Center	1 (2.04%)	1 (2%)			

The inclusion criteria are as follows [11]: (1) women aged between 40 and 80 years with unilateral disease; (2) patients who had not received neoadjuvant chemotherapy, endocrine therapy, radiotherapy, or targeted therapy before surgery; (3) patients who can undergo lymph node dissection; (4) high-grade intraductal breast carcinoma or invasive adenocarcinoma (non-special type) were pathologically diagnosed; and (5) complete clinical data.

The exclusion criteria are as follows: (1) patients with inflammatory breast cancer or invasive cancers, such as tubule carcinoma, apocrine adenocarcinoma, and lipid-rich carcinoma; (2) patients with other types of malignant tumors; (3) male breast cancer or occult breast cancer; (4) advanced breast cancer with distant metastasis; and (5) patients with incomplete clinical data, auxiliary examination, or pathological results from other hospitals. The clinical study was approved by the hospital ethics committee and performed in accordance with the Helsinki Declaration. The family members were informed and voluntarily signed the informed consent form.

### Method

The general data, color ultrasound characteristics, molybdenum targets, pathological features, and molecular pathology data of breast cancer patients were collected and collated by two investigators in the case system of our hospital. Subsequently, two investigators checked the data, including color ultrasound characteristics. The molybdenum targets and pathological features were determined by the Department of Ultrasound and Pathologists. The traditional blue dye method was used for sentinel lymph node biopsy tracing. For patients with micrometastasis, the scope of lymph node dissection was expanded, that is, dissection of axillary lymph nodes, subclavian lymph nodes, and intermuscular lymph nodes. The general data included age, tumor diameter, tumor number (single or multiple), tumor location (left or right), and the quadrant where the tumor was (outer upper, outer lower, inner upper, inner lower, and central regions). The color ultrasound data included whether the tumor margin was regular, whether there was a blood flow signal, and whether the posterior echo was attenuated, as well as the BI-RADS category. Molybdenum target features included regular tumor margins, presence of calcified foci, and BI-RADS category. Routine pathological features included histological grades (I, II, and III), pathological types (DCIS for ductal carcinoma and IDC for infiltrating adenocarcinoma), and molecular types (luminal A, luminal B, HER2 overexpression, and tri-negative). Routine pathology data also contained immunohistochemical staining for specific proteins across keratin 5/6 (CK5/6), cadherin E (E-cadherin), multidrug resistance gene 1 (MDR-1), epithelial growth factor receptor (EGFR), and keratin 19 (CK19). The expression score was based on the positive area of the protein (0–4) × staining intensity (0–3) [12, 13]. TP53, BRAC1/2, BRAF, ATM, and PALB2 were determined by first-generation sequencing.

### Statistical analysis

IBM SPSS 20.0, statistical software was used for data analysis. The measurement data that followed a non-normal distribution were represented as medians and subject to the Mann–Whitney *U* rank sum test. Enumeration data were expressed as [*n* (%)] and analyzed using the *χ*^2^ test. Logistic regression analysis was used for statistical analysis, and the measurement data were converted into binary counting data for calculation. All data were double-tailed with 95% confidence intervals, and statistical significance was set at *P* < 0.05. The receiver operating characteristic (ROC) curve was constructed using R language to draw the area under the curve (AUC). The closer the AUG value is to 1, the stronger is the accuracy of this indicator. AUC between 0.5 and 0.7 indicated general accuracy, while the value > 0.7 suggested strong accuracy [14].

## Result

### Univariate logistic regression analysis of general data

There was no statistically significant difference in tumor number, tumor location, or tumor quadrant between the two groups (*P* > 0.05). Age and tumor diameter were associated with sentinel lymph node metastasis (*P* < 0.05), as shown in Table 1.

### Univariate logistic regression analysis of color ultrasonography characteristics

There was no statistically significant difference in tumor margin, blood flow signal, and posterior echo attenuation between the two groups (*P* > 0.05), while the BI-RADS category was associated with sentinel lymph node metastasis (*P* < 0.05), as shown in Table 2.

**Table 2:** Univariate logistic regression analysis of color ultrasonography characteristics

Characteristic	Classification	Lymph node metastasis group (*n* = 49)	Non-lymph node metastasis group (*n* = 50)	*χ*^*2*^	*OR*	*P* value
Tumor margin (%)	Regular	4 (8.16%)	8 (16%)	1.37	1.04 (0.77~1.31)	0.13
	Irregular	45 (91.84%)	42 (84%)			
Blood flow signal (c%)	No	10 (20.41%)	12 (24%)	0.04	0.93 (0.81~1.07)	0.93
	Yes	39 (79.59%)	38 (76%)			
Posterior echo attenuation (%)	No	20 (40.82%)	24 (48%)	0.94	0.90 (0.55~1.10)	0.13
	Yes	29 (59.18%)	26 (52%)			
BI-RADS grade (%)	< 4	3 (6.12%)	6 (12%)	7.22	1.42 (1.23~1.69)	0.00
	4a	4 (8.16%)	9 (18%)			
	4b	10 (20.41%)	15 (30%)			
	4c	12 (24.49%)	13 (26%)			
	5	20 (40.82%)	7 (14%)			

### Univariate logistic regression analysis of molybdenum target characteristics

There was no statistically significant difference in tumor margin and calcification between the two groups (*P* > 0.05), whereas the BI-RADS category was associated with sentinel lymph node metastasis (*P* < 0.05), as shown in Table 3.

**Table 3:** Univariate logical regression analysis of molybdenum target characteristics

Characteristic	Classification	Lymph node metastasis group (*n* = 49)	Non-lymph node metastasis group (*n* = 50)	*χ*^*2*^	*OR*	*P* value
Tumor margin (%)	Regular	7 (14.29%)	14 (28%)	1.81	1.09 (0.83~1.21)	0.07
	Irregular	42 (85.71%)	36 (72%)			
Calcification (c%)	Without	26 (53.06%)	23 (46%)	0.94	1.01 (0.71~1.19)	0.20
	With	23 (46.94%)	27 (54%)			
BI-RADS grade (%)	< 4	6 (12.24%)	10 (20%)	6.23	1.32 (1.14~1.57)	0.02
	4a	9 (18.37%)	12 (24%)			
	4b	14 (28.57%)	15 (30%)			
	4c	11 (22.45%)	8 (16%)			
	5	9 (18.37%)	5 (10%)			

### The evaluation criteria of ER, PR, and HER in the groups

**Fig. 1:**
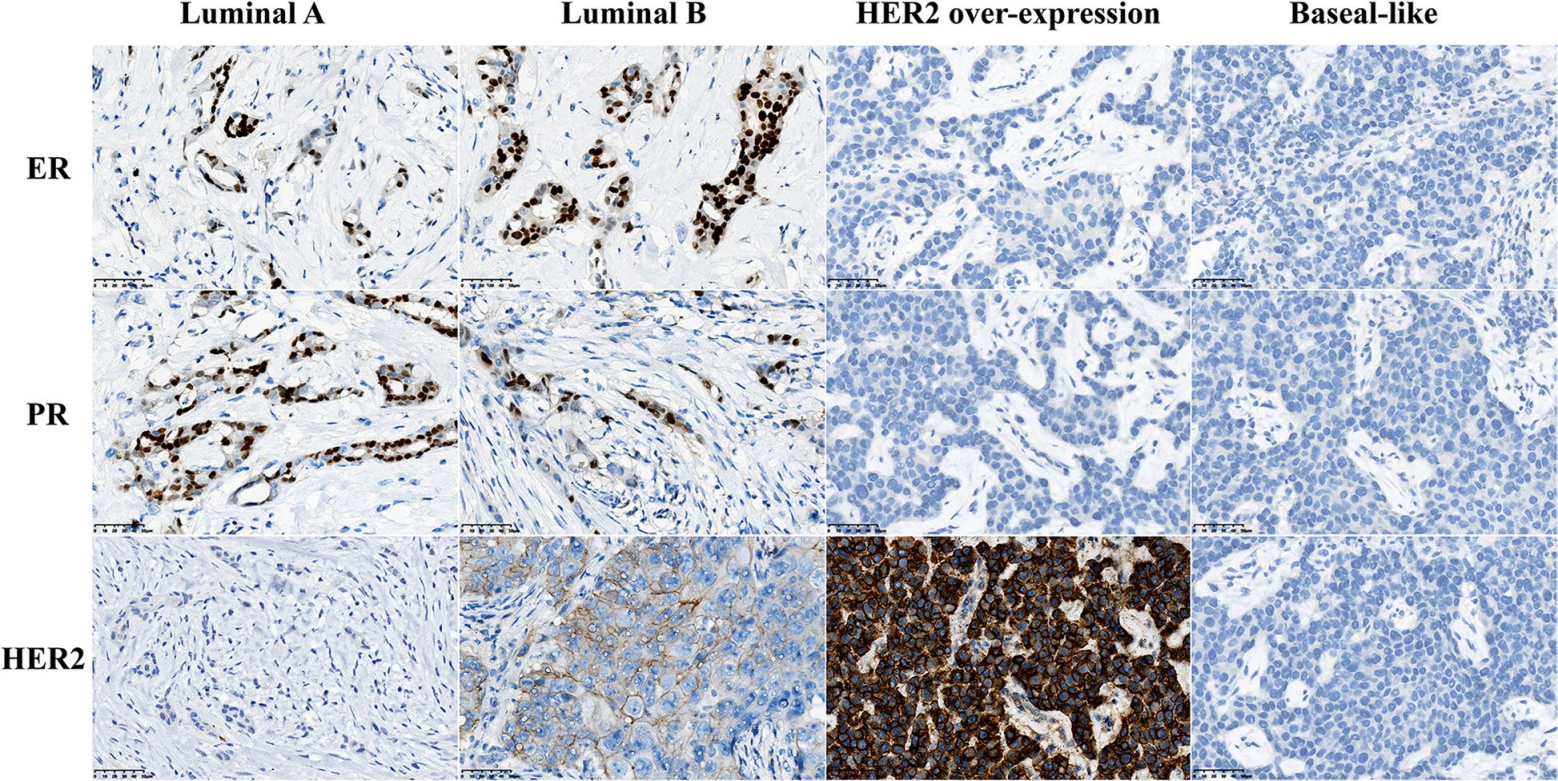
The evaluation criteria of ER, PR, and HER in the groups

In this study, ER (+), PR (++), and HER (−) were determined as the features of luminal A breast cancer, ER (+ − ++), PR (+), HER (+ − ++) as luminal B, ER (−/+), PR (−), and HER (+++) as HER2 overexpression in breast cancer, and ER (−), PR (−), and HER (−) as basal-like breast cancer, as shown in Fig. 1.

### Univariate logistic regression was used to analyze the routine pathological characteristics

There was no statistically significant difference in histological grade and molecular typing between the two groups (*P* > 0.05), while the pathologic type was associated with sentinel lymph node metastasis (*P* < 0.05), as shown in Table 4.

**Table 4:** Univariate logistic regression was used to analyze the routine pathological characteristics of the two groups

Characteristic	Classification	Lymph node metastasis group (*n* = 49)	Non-lymph node metastasis group (*n* = 50)	*χ*^*2*^	*OR*	*P* value
Histological level (%)	I	7 (14.28%)	10 (20%)	0.75	0.90 (0.72~1.09)	0.27
	II	32 (65.31%)	30 (60%)			
	III	10 (20.41%)	10 (20%)			
Pathological type (%)	DCIS	3 (6.12%)	11 (22%)	4.04	1.21 (1.06~1.40)	0.04
	IDC	46 (93.88%)	39 (78%)			
Molecular type (%)	Luminal A	11 (22.45%)	9 (18%)	2.06	0.93 (0.41~1.30)	0.07
	Luminal B	14 (28.57%)	10 (20%)			
	HER2 overexpress	16 (32.65%)	21 (42%)			
	Tri-negative	8 (16.33%)	10 (20%)			

### Special immunohistochemical evaluation criteria

**Fig. 2:**
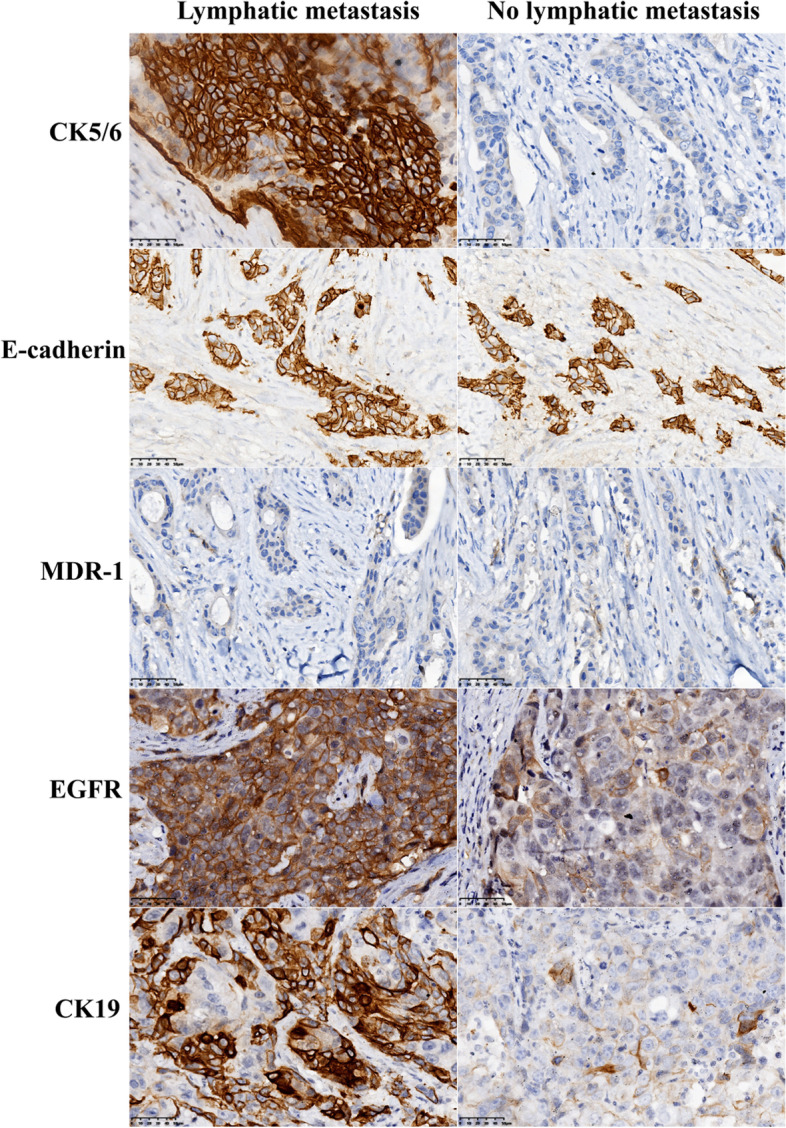
Special immunohistochemical evaluation criteria

In this study, the median CK5/6 score was 10 (9–12), median E-cadherin score 8 (6–11), median MDR-1 score 3 (1–6), median EGFR score 10 (8–12), and median CK19 score 9 (6–12) in the lymph node metastasis group. The corresponding figures in the other groups were 3 (1–6), 7 (5–11), 3 (1–5), 7 (4–10), and 3 (1-5), respectively (Fig. 2).

### Univariate logistic regression analysis of the special immunohistochemical characteristics

There was no statistically significant difference in E-cadherin and MDR-1 scores between the two groups (*P* > 0.05), while CK5/6, EGFR, and CK19 were associated with sentinel lymph node metastasis, as shown in Table 5.

**Table 5:** Univariate logical regression analysis of the special immunohistochemical characteristics

Characteristic	Lymph node metastasis group (*n* = 49)	Non-lymph node metastasis group (*n* = 50)	*Z*	*OR*	*P* value
CK5/6 (grade)	10 (9–12)	3 (1–6)	4.14	1.33 (1.10~1.52)	0.01
E-cadherin (grade)	8 (6–11)	7 (5–11)	0.75	1.01 (0.76~1.23)	0.29
MDR-1 (grade)	3 (1–6)	3 (1–5)	0.22	0.94 (0.68~1.23)	0.89
EGFR (grade)	10 (8–12)	7 (4–10)	3.84	1.29 (1.12~1.48)	0.04
CK19 (grade)	9 (6–12)	3 (1–5)	4.07	1.31 (1.17~1.56)	0.03

### Univariate logistic regression analysis of gene mutations

BRAF, ATM, and PALB2 levels were not significantly different between the two groups (*P* > 0.05). However, TP53 and BRAC1/2 were associated with sentinel lymph node metastasis (*P* < 0.05), as shown in Table 6.

**Table 6:** Univariate logistic regression analysis of gene mutations

Characteristic	Classification	Lymph node metastasis group (*n* = 49)	Non-lymph node metastasis group (*n* = 50)	*χ*^*2*^	*OR*	*P* value
TP53 (%)	Without	7 (14.29%)	1 (2%)	3.88	1.23 (1.04~1.39)	0.04
	With	42 (85.71%)	49 (98%)			
BRAC1/2 (%)	Without	6 (12.24%)	0 (0%)	3.80	1.21 (1.01~1.33)	0.04
	With	43 (87.76%)	50 (100%)			
BRAF (%)	Without	0 (0%)	2 (4%)	0.17	0.94 (0.82~1.04)	0.90
	With	49 (100%)	48 (96%)			
ATM (%)	Without	0 (0%)	0 (0%)	0.04	0.92 (0.90~0.95)	0.98
	With	49 (100%)	50 (100%)			
PALB2 (%)	Without	1 (2.04%)	0 (0%)	0.14	0.79 (0.68~0.89)	0.97
	With	48 (97.96%)	50 (100%)			

### Multivariate logistic regression analysis of risk factors for sentinel lymph node metastasis

Logistic regression analysis showed that age, tumor diameter, BI-RADS category, pathological type, CK5/6, EGFR, CK19, TP53, and BRAC1/2 were independent risk factors for sentinel lymph node metastasis of breast cancer (*P* < 0.05), as shown in Table 7.

**Table 7:** Logical regression analysis of multiple factors

Factors	*β*	*SE*	*Waldχ*^*2*^	*P* value	*OR* value	95%*CI*
Age	0.65	0.43	8.60	0.02	3.52	2.78~6.04
Tumor diameter	0.51	0.60	8.10	0.00	4.04	3.27~6.51
BI-RADS grade	0.73	0.28	7.04	0.03	3.10	2.33~5.28
Pathological type	0.53	0.63	6.94	0.04	3.07	2.50~4.92
CK5/6	0.60	0.46	8.01	0.02	3.37	2.99~5.78
EGFR	0.58	0.59	7.33	0.03	3.24	2.40~6.13
CK19	0.75	0.23	7.88	0.03	3.18	2.46~5.90
TP53	0.56	0.59	7.10	0.03	3.14	2.28~5.04
BRAC1/2	0.51	0.64	6.99	0.04	3.04	2.34~4.20

### ROC curve of independent risk factors for sentinel lymph node metastasis in breast cancer

**Fig. 3:**
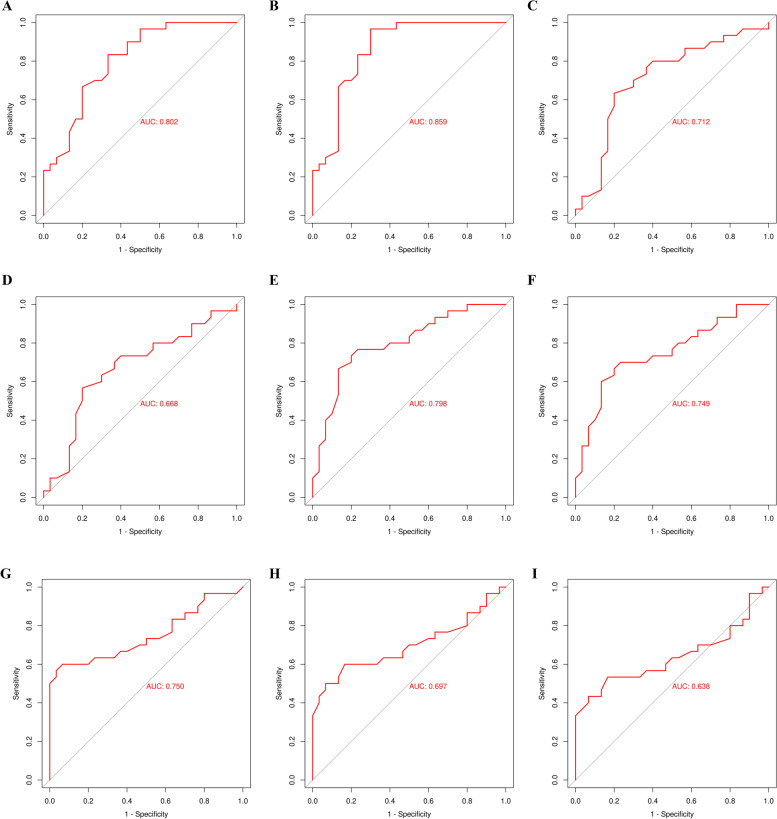
ROC curve of independent risk factors for sentinel lymph node metastasis in breast cancer. **A** Age, **B** tumor diameter, **C** BI-RADS grades, **D** pathological type, **E** CK5/6, **F** EGFR, **G** CK19, **H** TP53, and **I** BRAC1/2

The area under the ROC curve (AUC) was observed in the two groups with respect to age, tumor diameter, BI-RADS grade, pathological type, CK5/6, EGFR, CK19, TP53, and BRAC1/2 for sentinel lymph node metastasis (Fig. 3).

The “Multivariate logistic regression analysis of risk factors for sentinel lymph node metastasis” and the “ROC curve of independent risk factors for sentinel lymph node metastasis in breast cancer” sections indicate that age, tumor diameter, BI-RADS score, pathological type, CK5/6, EGFR, CK19, TP53, and BRAC1/2 are independent risk factors affecting sentinel lymph node metastasis of breast cancer, which may provide some guidance on sentinel lymph node metastasis.

## Discussion

According to the latest epidemiological data, the incidence of breast cancer in China is as high as 42.57/100,000, accounting for 19.2% of all malignant tumors in women. It has surpassed lung cancer as a malignant tumor with the highest incidence [1, 15]. For treatment, surgical operations are clinically preferred, including radical mastectomy, breast-conserving surgery, and expanded radical mastectomy [16]. Axillary lymph node dissection, an important procedure in breast cancer surgery, is of great importance in determining the pathological status, stage, and prognosis of breast cancer [17]. However, it is relatively traumatic, causes serious complications such as upper extremity lymphedema, sensory numbness, or limited shoulder joint motion, and may affect the prognosis of patients [3, 18]. Sentinel lymph nodes are the first lymph nodes that are passed through in the process of tumor metastasis in the drainage area of the primary tumor. They can be extracted through a small incision after contrast agent intervention with high accuracy, gradually replacing axillary lymph node dissection as the “gold standard” of lymph node biopsy for breast cancer surgery [4, 19]. And De Luca et al. reported that cyanoacrylate glue in association with closed suction axillary drain seems to contribute to the reduction in days of axillary drain permanence and of postoperative infections and prevent seroma after axillary dissection in patients with breast cancer [20]. Tests on sentinel lymph nodes are minimally invasive and accurate in assessment and can effectively reduce surgical trauma in patients with lymph node metastasis. Among the available detection techniques, Doppler ultrasound detection and molybdenum target detection are highly sensitive [6, 21].

Chen et al. [22] pointed out that age is directly related to sentinel lymph node metastasis and may be linked to poor immune function in elderly patients. In this study, the age of the metastasis group was higher than that of the other groups. The odds ratio (OR value was 1.24 (1.07–1.41) and positively correlated with metastasis, which was consistent with the results of previous studies [23]. Moreover, this study also found that the larger the tumor diameter, the higher the sentinel lymph node metastasis rate, which may be related to the larger cell base of the tumor tissue and the stronger invasion of the surrounding vessels [24].

Among the influencing factors analyzed using color Doppler ultrasonography and molybdenum target characteristics, BI-RADS grade was directly related to sentinel lymph node metastasis, possibly because the higher the ultrasound grade, the higher the clinical stage of breast cancer, and the higher the probability of sentinel lymph node metastasis. This is related to the biological behavior of breast cancer. However, the regularity of the tumor margin, blood flow, and calcification foci did not affect lymph node metastasis.

Pathological characteristics can directly affect the proliferation and metastasis of malignant tumors, in which the degree of differentiation, growth pattern, and biological behavior of tumor cells can be used as important indicators to evaluate the clinical stage and prognosis of breast cancer [25]. In this study, pathological types were correlated with sentinel lymph node metastasis because intraductal carcinoma is carcinoma in situ and has a low probability of lymph node metastasis. Moreover, invasive carcinoma has a poor growth pattern and biological behavior, resulting in a higher probability of lymph node metastasis. In addition, there were no statistically significant differences in histological grade and molecular typing between the two groups. Molecular typing is directly related to the prognosis of breast cancer patients, especially basal-like breast cancer, whose survival rate is far lower than that of other types [26]. However, in terms of sentinel lymph node metastasis, there was no statistical difference in this study, and more clinicians are needed to conduct studies with larger samples.

The expression of breast cancer-related proteins plays a key role in the occurrence and development of breast cancer. CK5/6, E-cadherin, MDR-1, EGFR, and CK19 are common immunohistochemical indicators and drug resistance indicators [27–30]. CK5/6 and CK19 are the most widely used immunohistochemical markers of tumor cell epithelium. In this study, we quantified the expression of these indicators and found that the stronger the expression of epithelial markers, the higher the probability of sentinel lymph node metastasis. EGFR is a transmembrane protein directly related to the proliferation, metastasis, and apoptosis of tumor cells and therefore can affect the metastasis of sentinel lymph nodes, which is consistent with the results of previous studies [31].

Gene mutations are the focus of clinical research on the prognosis of breast cancer. In this study, the mutation rates of TP53, BRAC1/2, BRAF, ATM, and PALB2 in patients were statistically analyzed using first-generation sequencing. In this study with a limited sample size, it was found that TP53 and BRAC1/2 may affect sentinel lymph node metastasis; however, validation with a larger sample size is needed.

## Conclusions

In this study, we identified independent risk factors for sentinel lymph node metastasis in breast cancer based on general data, color ultrasound, molybdenum, and pathological features. This indicates that color ultrasonography, molybdenum target, and pathology techniques can be used as important diagnostic and prediction methods for sentinel lymph node metastasis of breast cancer and can be used to build a prediction model, serving as a guide for Chinese community hospitals. In conclusion, age, tumor diameter, BI-RADS grade, invasive type, expression of CK5/6, EGFR, and CK19, and mutations in TP53 and BRAC1/2 were positively linked to sentinel lymph node metastasis of breast cancer as independent risk factors. Clinical studies should focus on these risk factors in order to strengthen the management and control of sentinel lymph node metastasis in high-risk breast cancer and perform better in early chemotherapy, radiotherapy, or targeted therapy.

## Data Availability

The datasets used and/or analyzed during the current study are available from the corresponding author on reasonable request.
